# The institutional and socio-technical determinants of renewable energy production in the EU: implications for policy

**DOI:** 10.1007/s40812-022-00212-6

**Published:** 2022-03-01

**Authors:** Alessandro Marra, Emiliano Colantonio

**Affiliations:** University d’Annunzio of Chieti and Pescara, Pescara, Italy

**Keywords:** Renewable energy production, Environmental policy, Public awareness, Lobbying, Education, Income, Energy imports, Q40, Q28

## Abstract

Despite the consensus that the transition to renewable energy is a process that encompasses institutional, regulatory, technical, political, social, and cultural aspects, such issues have rarely been addressed in a comprehensive way. This study explores the determinants of renewable energy production (REP), focusing on institutional and socio-technical aspects. We employ a panel vector autoregressive (PVAR) model to test dynamic relationships for the period 1990–2015 among several variables, as have emerged in the literature: REP, policy stringency, public awareness, lobbying, education, controlling for income and energy imports. Focusing indiscriminately on 18 European Union (EU) member states, the results show that environmental policy stringency does not influence REP, while income and education impact negatively. This evidence is counter-intuitive, and would be surprising if we did not consider the strong heterogeneity between countries. EU member states are engaging in energy transition at different speeds, depending on their individual starting point: this differs from country to country in terms of installed capacity and energy security. Moving from the recent European Green Deal, we divide the sample into two panels based on energy imports to account for different starting points: countries less active on the production side (that depends particularly on energy imports), and countries more active on the production side. Results for the first panel show that an increase in policy stringency would lead to a decrease in lobbying and an increase in REP. Policy efforts must be clearly established and consistently preserved to support REP, at least if there are increasing returns to exploit. Results for the second panel show that lobbying negatively affects the transition to REP, while an increase in public awareness will promote an increase in REP. Therefore, priority should be given to the ‘social’ aspect, and policymakers should increase efforts to reduce the proportion of energy generated from oil, natural gas, coal, and nuclear fuel.

## Introduction

Renewable energy sources are emerging as an important component with regard to meeting global energy demand. According to the International Energy Agency (IEA, 2019), the capacity for renewable power will increase by 50% in the coming years. Solar photovoltaic energy accounts for 60% of the expected growth, onshore wind accounts for 20%, and offshore wind for only 4%. The last of these is expected to triple in capacity by 2024, thanks to competitive auctions in the European Union (EU) and market expansion in China and the United States (US). In addition, bioenergy capacity is expected to increase, especially in China, India, and the EU. The increase in hydropower will decelerate, although it will still account for 10% of the growth in renewable energy (IEA, 2019). In 2020, the deployment of renewable energy for generating electricity has increased by 7%: the Covid-19 crisis has not halted its growth (IEA, 2020).

In the EU, primary renewable energy production (REP) increased by 49% between 2008 and 2018. The most important source was wood and other biofuels, which accounted for more than 40%. Wind power accounted for 14% of the total, confirmed as the second main source, followed by hydro power with 11% of the total. Despite low levels of production, the output of biogas, liquid biofuels, and solar energy increased fast, accounting for a 7%, 7%, and 6%, respectively of the EU’s REP (EC, 2021). Although the installed capacity for REP is increasing, considerable differences remain within the EU. In 2018, renewable energy shares varied considerably across EU member states, ranging from 15 to 100%. Such differences are expected to continue because of the persistence of country-specific conditions, different starting points, and existing policies (IRENA, 2018).

A growing literature has emerged to identify the factors that prompt renewable energy at the national level (Gan & Smith, 2011; Marques & Fuinhas, 2011). However, the understanding of its determinants remain incomplete, due to the use of different methodologies and frameworks, and to the lack of a comprehensive focus on institutional and socio-technical issues (Bourcet, 2020; Can Şener et al., 2018).

First, there is limited understanding regarding the determinants of REP, even though some agreement has emerged on a couple of mechanisms: regulatory and environmental policies have a positive effect, while lobbying in favour of traditional energy sources produces a negative effect. Secondly, despite the consensus that the transition to renewable energy is a process that encompasses institutional, regulatory, technical, political, social, and cultural aspects, such issues have been rarely addressed in a comprehensive way in the literature (Andrews-Speed, 2016; Sovacool, 2009).

Some energy companies still reject renewable resources because they prefer big and conventional power plants. Many consumers do not fully appreciate the benefits deriving from more renewable systems in terms of the reduction of carbon dioxide emissions. Sometimes public authorities are not able to set up the ‘right’ system or provide the ‘right’ incentives to encourage more renewable production. In such circumstances, institutional and socio-technical issues act as impediments and barriers to renewable deployment. Nonetheless, such aspects need to be adequately understood to realize how renewable energy is going to expand in the future: while technological advances are important, resistance from firms, consumers and public authorities can impede or slow the transition considerably (Smith et al., 2005). Improved understanding of such institutional and socio-technical factors will allow scholars and policymakers to provide forecasts for renewable energy that are better grounded in the socio-political context (Andrews-Speed, 2016).

Today, compared to a few years ago, we appreciate to a greater extent the increased public awareness of the dangers of climate change, the desirability of using renewable energy, the growing adoption of environmental policy measures and incentive schemes to boost clean energy deployment, and the common acceptance of the green paradigm by both large multinationals and small and medium-sized enterprises.

All this calls for a need to revisit the determinants of REP and a need to focus on the socio-technical aspects. The purpose of the study is to investigate the determinants of REP in different EU member states while controlling for income and energy imports, focusing on several institutional and socio-technical aspects in the form of regulatory constraints and policy stringency, private interests and lobbying, environmental pollution and public awareness, technical understanding and the level of education. We employ a panel vector autoregressive (PVAR) model in first differences to test the complex dynamic relationships among the above variables for the longest time span possible—from 1990 to 2015—for 18 EU member states.

Although there are some differences, all EU member states are expected to raise the level of production from renewable sources by employing several levers. Nonetheless, the results with regard to the entire sample of countries show that environmental policy stringency does not influence REP, while income and education impact negatively. This evidence is counter-intuitive and would be surprising if we did not consider the strong heterogeneity between countries. EU member states are engaging in a transition to REP at different speeds, based on their starting point: this differs from country to country in terms of installed capacity and energy security. More specifically, countries with relatively higher energy imports and lower installed capacity are expected to act more quickly, since they face lower obstacles to initiating a more vigorous transition to renewable energy (Kahia et al., 2017).

This same perspective has been adopted by the European Commission in the recent European Green Deal (EC, 2019). Accordingly, as suggested in the literature (Marques & Fuinhas, 2012), we divide the sample into two panels, using the share of energy imports to account for the different starting points. Energy imports, combining installed capacity and energy security issues, properly condenses differing policy, strategic and industrial choices, and allows us to distinguish between countries that are less active in terms of production (depending on energy imports from third countries; panel A, high energy importers) and countries that are more active (panel B, low energy importers).

The remainder of the paper is organized as follows. Section 2 reviews the literature on the determinants of REP. Section 3 presents the empirical analysis, including the model specifications and the methodology used (Sect. 3.1), the testing framework (Sect. 3.2), and the results (Sects. 3.3 and 3.4). Conclusions and policy implications based on the findings are presented in Sect. 4.

## Literature

The transition to renewable energy involves deep changes in society at different levels. Most of these changes can be framed within institutional theory, according to which institutions set the pace and the direction of economic and political development (Blyth, 2002; North, 1990, 2005). In the following review we intend to emphasise some key points in the literature on renewable energy in terms of institutional theory. Institutions, such as policy and regulation, exert a direct and noteworthy effect on renewable deployment. Secondly, analyses of policy and regulations need to consider path dependence as an indispensable element of transition processes. Lastly, institutions cannot be viewed as being isolated from the underlying socio-technical substratum that generates them: governments are often necessary elements but are not sufficient in terms of these kinds of transitions.

In this section, we do not intend to provide a comprehensive overview of how institutional theory can help rationalise the energy transition, because such an attempt would go beyond the scope of the paper. In effect, institutions have been variously conceptualised, and there exist several strands of theory which need to be investigated (Andrews-Speed, 2016). We refer to further and more detailed literature on this theme (Nilsson et al., 2011; Smith et al., 2005).

Since the transition to renewable sources is a socio-technical process, we need to start somewhere to differentiate between institutional, regulatory, technical, political, social, and cultural aspects, even if any attempt to divide the technical from the political, or even the social from the technical is complicated, and misses the point that such aspects are fundamentally integrated. We focus on the main factors that, according to the literature, play a role in guiding the transition: regulatory constraints and policy stringency, environmental pollution and public awareness, private interests and lobbying, technical understanding and level of education, and controlling for income and energy imports.

The choice of the main determinants accounted for, and the proxies used in the empirical analysis, are coherent with the extant literature.

There is little doubt about the impact of regulatory constraints and policy stringency on the environment, even though there is no clear consensus on how to measure such aspects. Bird et al. (2005) contended that it is difficult to discern one single determinant of wind power development. Some of the determinants observed in 12 US Federal States include policy aspects such as renewable portfolio standards (RPS, a regulation that requires an increase in REP), federal and state financial incentives, and market drivers. More specifically, RPS appear to be the most successful. Menz and Vachon (2006) examined the policies adopted at state level to promote renewable energy. They found a substantial disparity among states in terms of the renewable power policies they adopt. Shrimali and Kniefel (2011) used data from over 50 US states for the time interval 1991–2007 and a model with state-specific trends to investigate the consequences of state policies on the diffusion of various renewable sources. RPS, with specific requirements in terms of capacity and sales, was found to generate significant effects on renewable energy deployment. Polzin et al. (2015) examined the consequences of public policy measures on renewable energy investment in capacity. The authors investigated the impact of different policies in OECD countries to identify an effective mix that could boost renewable energy. The findings suggest the need for policies that take account of market conditions and technological development. To increase investment, recommended policies include economic and fiscal incentives, specifically for new and emerging technologies. All the results reinforce the expected positive relationship between regulation and REP.

Composite indicators such as the Environmental Policy Stringency (EPS) index, have been largely used to explore the advantage of incorporating market-based and non-market-based regulations (Georgatzi et al., 2020; Ouyang et al., 2019). Although we could have relied on individual indicators such as feed-in tariffs, trading schemes, and taxes within the composite index, we opted for a more inclusive measure: the EPS index combines 14 different indicators into one single, comparable, country-specific proxy of policy stringency. Moreover, we checked for positive and significant correlations between market-based and non-market-based dimensions (0.53), and between each dimension and the composite index, and obtained 0.83 and 0.93, respectively (Hille & Möbius, 2019).

As expected, income (taken as GDP per capita) is the economic variable most frequently used to explain renewable energy deployment. There is much debate as to whether or not, at least in advanced countries, higher income can be positively associated with higher energy production, both from renewable and non-renewable sources. The GDP effect has been tested recurrently in the literature (Alper & Oguz, 2016; Menegaki, 2011; Narayan & Smyth, 2008; Sadorsky, 2009a, 2009b). A greater income level is expected to be associated with greater renewable energy for two reasons: first, greater GDP implies more resources being available to implement and promote renewable energy; second, a greater income level implies greater potential to support higher policy costs (Chang et al., 2009). Conversely, Marques and Fuinhas (2011) found that income and the price of fossil-based fuels were not major factors in the deployment of renewable energy sources. Marques and Fuinhas (2012) analysed the relationships between energy sources and economic growth for 24 EU countries between 1990 and 2007, and suggested that the consequence of renewable deployment replaces the impact of generating income by using natural resources sourced locally. The major costs associated with supporting renewable sources tend to place an excessive burden on the economy, due to rising electricity tariffs.

Policy and economic variables have been used within more comprehensive frameworks to understand their role and scope. Carley (2009) appraised the implementation of RPS policy achieved by state energy programs working on a larger spectrum of variables: the author found that institutions, natural resources, deregulation, GDP per capita, electricity use per capita, electricity prices, and the presence of regional RPS were all related to renewable energy. Gan and Smith (2011) identified key factors that might drive different shares of renewable energy in terms of the energy supply among OECD countries. They found that, in addition to country-specific factors, GDP, along with renewable energy deployment policies, has a positive effect on the per capita supply of renewable energy. R&D expenditure, energy prices, carbon-dioxide emissions, and other energy policies had no significant effect on renewable energy. Nonetheless, this does not necessarily mean they are not potential drivers; instead, it implies they were not of a magnitude sufficient to appreciably affect energy supply between 1994 and 2003.

Following Carley (2009) and Sadorsky (2009a), most of the literature introduced an environmental indicator in the form of carbon-dioxide emissions as a proxy for environmental concerns and degradation (Bourcet, 2020). Carbon dioxide emissions are positively associated with REP (Menegaki, 2011; Sadorsky, 2009a; Salim & Rafiq, 2012; van Ruijven & van Vuuren, 2009). This may be explained by the fact that a high level of carbon dioxide emissions causes an increase in the demand for environmental protection. Nonetheless, Marques and Fuinhas (2011) showed that public awareness of environmental issues and carbon dioxide targets are not sufficient in themselves to encourage a shift to renewable energy. Citizens should carry the initial economic costs by supporting policy costs such as paying higher prices and taxes.

Likewise, the role that the dependency on fossil fuels and the power of lobbies might play in balancing out environmental policies has been widely debated (Marques & Fuinhas, 2011; Marques et al., 2010). Marques et al. (2010) showed that lobbying for fossil sources reduces renewable use. Marques and Fuinhas (2011) explored the commitment to renewable energy in 24 EU member states, and showed that non-renewable energy sources restrain the stimulus towards renewable energy. The development and growth of renewable energy can be prevented by interest groups, including trade associations associated with the fossil and nuclear energy sectors, which could increase the percentage of fossil- and nuclear-based energy. The ease with which fossil resources can be stored, and the already-installed capacity, might justify why the deployment of renewable energy is hampered. Since the effect of lobbying is suggested to be a major determinant (Huang et al., 2007; Sovacool, 2009), we opted for including such a dimension in the analysis.

It is commonly accepted that energy imports are inversely correlated with the installed capacity of traditional energy sources and, consequently, have negative repercussions on the industrial choices and strategies that can be pursued in terms of renewable energy production (Kahia et al., 2017). The share of energy imports is, at the same time, a starting point and an industrial policy lever with regard to ensuring energy self-sufficiency and security (Narbel, 2013; Valdés Lucas et al., 2016). Even though there is consensus in recognizing a negative association between energy imports and the deployment of renewable energy (Marques et al., 2010), this relationship is not always empirically confirmed (Marques & Fuinhas, 2012).

To better discern the underlying relationships between the above discussed socio-technical variables, we added a further interpretative element by introducing the variable education in the sense that this can generate reinforcing mechanisms to the advantage of the environment and renewable sources (Xie et al., 2017).

Vachon and Menz (2006) examined the impact of social, political, and economic interests on the propensity to implement renewable energy policies. The results suggest that social interests, measured by income, education, and involvement in environmental lobbying, were associated with the adoption of green policies. Furthermore, the authors explained that an understanding of the advantages of environmental policies is positively correlated to levels of education for several reasons, including a more cogent appraisal of the pros and cons of different policies, and a greater perception of environmental issues. Huang et al. (2007) explored the relationship between education, policy, and renewable energy. Higher education was found to be positively associated with the awareness of the pernicious effects of fossil fuel use and, albeit less relevant, political problems deriving from a high reliance on oil imports. We expect this variable to have a positive impact on the adoption of renewable-oriented policies.

A negative relationship is thought to exist between environmental pollution and education levels: exposure to higher education facilitates the formation of social awareness and solutions to common problems (Bimonte, 2002; Dasgupta et al., 2016). It is interesting to notice how education, proxied by the ratio of total enrolment in third-level or post-secondary education (Wang & Shao, 2019), is referred to as ‘informal’ regulation, given the role it plays in strengthening the environmental awareness of a society (Neves et al., 2020). An understanding of the benefits derived from environmental policies is positively connected with education for several reasons. These include a more appropriate evaluation of the benefits and costs of various regulatory measures, greater attention to environmental problems such as carbon dioxide emissions, and so on (Zarnikau, 2003). However, higher education might also promote more effective lobbying in favor of non-renewable sources, if we read such a relationship in a specular manner to the role that lack of expertise among consultants, experts and other professionals is expected to play in the adoption of renewable sources (Gan et al., 2007).

According to some scholars, the prices of conventional energies such as natural gas, oil, coal, and nuclear power might also play a role (van Ruijven & van Vuuren, 2009). Given the degree of substitution of different energy sources, it might be expected that higher-priced traditional resources imply greater use of renewable energy (Henriques & Sadorsky, 2008; Sadorsky, 2009a; Salim & Rafiq, 2012; Silk & Joutz, 1997). Nonetheless, if this is true, it must be acknowledged that such prices might influence consumption, and not the production of renewable energy, which is the focus of this article. The prices we need to consider when assessing the energy and industrial policy choices of a given country, should be the production or import prices of natural gas, oil, coal, and nuclear power to account for their effects on national renewable energy production. As is well known, such prices are not country-specific, and referring to international benchmarks and markets would not have been suitable for our purpose.

To sum up, although all the articles noted above consider one or more of our variables, they seem to lack a comprehensive approach: a limited number of socio-technical variables, the absence of a clear and unambiguous focus on renewable energy deployment, and an incomplete account about the existing differences across countries with respect to their institutional and socio-technical context. As a result, the very understanding of the determinants of REP remains limited (Bourcet, 2020; Can Şener et al., 2018). More specifically, it emerges that regulatory and environmental policies have a positive effect on renewable energy deployment, whereas lobbying on the part of traditional energy sources has a negative effect. Less convincing results accompany public awareness of environmental issues, which seems to be insufficient to promote a move towards renewable energy, and education, which can help only if it can support a better understanding of the advantages of green policies and renewable energy. Income levels should play a positive role, while evidence on the effects of energy imports is mixed. Overall, such effects should not overlook the importance of a country’s initial conditions and the role of path dependence.

## Empirical analysis

### Model specification and methodology

Based on the literature, we constructed a model focused on the following variables: renewable electricity production (% of total electricity output, REN); environmental policy stringency (EPS); carbon dioxide emissions (metric tons per capita, CO_2_); electricity production from oil, gas, coal and nuclear sources (% of total, OGCN); educational attainment level of the population (in %, EDU); gross domestic product per capita (constant 2010 US$, GDP); and energy imports (net, % of energy use, IMP).

As mentioned previously, REN denotes the share of renewable electricity production as a percentage of total electricity output, and is used as a proxy for REP. Many authors employ the share of renewable energy sources in electricity production as a dependent variable (Bourcet, 2020). To measure the role of regulatory constraints and policy stringency we adopt the variable EPS: it is an internationally-comparable index of the stringency of environmental regulation at the country level. Stringency represents the level to which environmental policies place an explicit or implicit price on environmentally-harmful behavior or pollution. The measure varies from 0 (not stringent) to 6 (the highest degree of stringency): it is built on the level of stringency of 14 environmental policy tools, mainly related to climate and air pollution (Botta & Koźluk, 2014; de Serres et al., 2010). Environmental pollution and public awareness are proxied by CO_2_: it measures carbon dioxide emissions generated during gas flaring, and as a result of the consumption of solid, liquid, and gas fuels. To consider private interests and lobbying, we adopt the variable OGCN. OGCN refers to electricity production from oil, gas, coal, and nuclear sources: larger consumption needs can be supplied by a combination of non-renewable and renewable energy sources. We controlled for the production of energy, expecting either a positive or negative effect on REN. OGCN might also refer to lobbying (Marques & Fuinhas, 2011, 2012; Marques et al., 2010), which creates a potential barrier to renewable energy. EDU is a proxy for technical understanding and the level of education. It measures the educational attainment level (upper secondary, post-secondary non-tertiary and tertiary education) of the population from 15 to 64 years. GDP is per capita gross domestic product, converted to international dollars using purchasing power parity rates. Data are in constant 2010 US dollars, converted from domestic currencies using 2010 official exchange rates. Finally, IMP can be considered a valuable proxy for the level of installed capacity and energy security.

The purpose of the paper is to examine the role of institutional and socio-technical variables in the production of renewable energy in 18 EU member states (Austria, Belgium, Czech Republic, Denmark, Finland, France, Germany, Greece, Hungary, Ireland, Italy, Netherlands, Poland, Portugal, Slovak Republic, Spain, Sweden, and the United Kingdom). Based on annual data from 1990 to 2015, we employed the PVAR approach, which allows us to highlight bidirectional dynamic effects and potential path dependences. Table 1 presents the variables, their definitions, and sources.

**Table 1:** Description of variables

Variable	Definition	Source
REN	Renewable electricity output (% of total electricity production)	World Bank
EPS	Environmental Policy Stringency Index	OECD
GDP	GDP per capita (constant 2010 US$)	World Bank
CO_2_	CO_2_ emissions (metric tons per capita)	World Bank
OGCN	Electricity production from oil, gas, coal and nuclear sources (% of total)	World Bank
EDU	Population 15–64 years by educational attainment level (%) (isced 3–8)	Eurostat
IMP	Energy imports, net (% of energy use)	World Bank

In line with Love and Zicchino (2006), the following PVAR model was used to estimate endogenous relationships among the variables described above:

1 $$X_{it} = f_{i} + \Gamma ( L )X_{it} + \varepsilon_{it} ,$$

where $X_{it}$ is the vector of stationary indicators, $f_{i}$ is the vector of country-specific effects, $\Gamma ( L)$ is a matrix whose elements are polynomial in the lag operator, and $\varepsilon_{it}$ is the random perturbation (later *d* denotes first difference operator). Table 2 provides the descriptive statistics.

**Table 2:** Descriptive statistics

Variable	Mean	Std. Dev.	Min	Max
REN	19.14	18.94	0.58	81.06
EPS	1.85	0.90	0.35	4.13
GDP	32.49	13.97	5.51	65.43
CO_2_	8.26	2.15	4.24	13.72
OGCN	79.25	19.14	17.72	99.28
EDU	64.42	15.68	19.30	87.60
IMP	48.92	30.04	− 65.69	90.68

### Testing framework

Variables at the macroeconomic level are often non-stationary, and this may generate spurious results in the econometric analysis. In such a context, first-difference transformation can be employed to solve the problem. Therefore, we conducted various first–second unit root tests to investigate the stationarity of the indicators. Specifically, IPS tests, MW tests, and Pesaran tests were employed to analyse the order of integration of our variables, checking the null hypothesis that assumes the presence of a unit root in the panel. The results show that, at conventional levels of significance, all variables are non-stationary in levels, but stationary after the first difference transformations (see Tables 3 and 4).

**Table 3:** Unit root tests: variables in level

Variable	IPS W-t-bar	MW	Pesaran Z-t-bar
REN	12.635	13.479	0.611
EPS	3.742	10.896	− 0.164
GDP	2.541	16.302	− 0.924
CO_2_	4.714	8.048	− 3.241***
OGCN	11.225	12.699	− 0.37
EDU	1.432	19.855	1.799
IMP	− 0.209	43.779	− 0.311

*p < 0.1; **p < 0.05; ***p < 0.01

**Table 4:** Unit root tests: variables in first differences

Variable	IPS W-t-bar	MW	Pesaran Z-t-bar
dREN	− 11.138***	224.079***	− 8.791***
dEPS	− 16.452***	204.742***	− 5.431***
dGDP	− 9.174***	145.072***	− 1.546***
dCO_2_	− 17.359***	178.240***	− 8.835***
dOGCN	− 11.883***	243.187***	− 8.882***
dEDU	− 15.690***	288.347***	− 7.019***
dIMP	− 13.026***	181.071***	− 6.343***

*p < 0.1; **p < 0.05; ***p < 0.01

To take account of cross-section interdependence, we conducted the cointegration tests introduced by Westerlund (2007). These tests are based on the null hypothesis of the absence of cointegration. None of the four tests allow us to reject the null hypothesis of absence of cointegration (see Appendix 1, Table 9). An estimation in first differences is therefore necessary since the variables in level are non-stationary and non-cointegrated.

The lag order selection was the final preliminary step in our analysis. Specifically, according to the econometric literature, the optimal lag length should minimize the moment model selection criteria MBIC, MAIC, MQIC proposed by Andrews and Lu (2001). Based on the three model selection criteria, a first-order PVAR was the chosen model (see Appendix 1, Table 10).

The deterministic fixed effects $f_{i}$ in Eq. (1) were removed by using the first difference transformation. As is well known, this method however may cause the so-called Nickell bias (Nickell, 1981), with the possibility of obtaining biased and inconsistent estimates using OLS (Baltagi, 2008). To overcome this difficulty, we used forward mean-differencing, also referred to as the Helmert transformation (Arellano & Bover, 1995; Love & Zicchino, 2006), which preserves the orthogonality between lagged regressors and transformed variables. The model may therefore be estimated using the Generalized Method of Moments (GMM) and the lagged values of the regressors can be used as instruments.

### Empirical results and discussion

After conducting the preliminary tests, the PVAR was estimated by selecting one lag and using the GMM-style option. This guarantees more efficient estimates by replacing missing values with zero (Holtz-Eakin et al., 1988). The results are presented in Table 5.

**Table 5:** PVAR results

Independent variables	Dependent variables						
	dREN	dEPS	dGDP	dCO_2_	dOGCN	dEDU	dIMP
dREN	− 4.470***	0.104***	0.006	0.041	4.233***	0.210***	0.984***
dEPS	− 0.577	0.069	1.271***	0.897***	− 0.461	1.868***	3.151***
dGDP	− 1.489***	0.086***	0.749***	0.280***	1.342***	0.297***	1.221***
dCO_2_	1.231**	− 0.398***	0.497***	− 0.430***	− 1.382**	− 0.267**	− 5.971***
dOGCN	− 4.208***	0.119***	− 0.005	0.054**	3.964***	0.224***	1.139***
dEDU	− 1.009***	0.007	0.093***	0.127***	1.083***	− 0.123*	0.269**
dIMP	0.104**	0.004	− 0.012	0.004	− 0.075	− 0.060***	0.352***

*p < 0.1; **p < 0.05; ***p < 0.01

The stability of the model was analysed and verified as the eigenvalues are strictly less than 1 (see Appendix 1, Table 11 and Fig. 2). This also shows that the employed variables are stationary (Lutkepohl, 2005). We also executed the Granger causality test which confirmed the existence of endogeneity (see Appendix 1, Table 12).

The results show that EPS does not seem to have, at least immediately, a significant impact on REN. This might be due to the strong heterogeneity across countries. If GDP rises, then REN decreases: economic growth is likely to increase the demand for energy, while renewables (especially in the past) offered only a modest contribution. This is also confirmed by the fact that, if GDP increases, OGCN will follow. An increase in CO_2_ and public awareness will promote an increase in REN. On the other hand, OGCN seems to negatively affect the transition to renewable energy. In other words, the existing energy production systems, mainly based on oil, natural gas, coal, and nuclear sources, could represent a substantial burden on the deployment of renewable energy. Our findings show that EDU significantly influences REN negatively. This does not necessarily mean that education is not important, or that it even might represent an obstacle to the energy transition. Two reasons can explain such an outcome. First, the observed countries with a higher level of education typically adopt models of production and consumption that inevitably need more energy, which means more carbon-dioxide emissions. Education (through public awareness) cannot compensate for such high levels of energy demand. In the long term, considering the correlation between the average values of EDU and CO_2_ for the time interval, we obtain positive values (above 0.70) except for Hungary, Sweden, and the Slovak Republic. This is in line with the literature on the effect of socio-economic and behavioral aspects on carbon dioxide emissions: education and CO_2_ emissions are positively correlated, but higher levels of education ‘reduce’ emissions when further factors are controlled for (Baiocchi et al., 2010; Büchs & Schnepf, 2013). Secondly, despite the importance of public awareness in the transition process, stakeholders might not exert enough pressure towards renewable sources. Finally, the increase in energy imports seems to push countries towards renewable sources. If GDP increases, there is room for more stringent environmental policies. If OGCN increases, the response is usually an increase in EPS. A more vigorous environmental policy, in turn, does not slow down economic growth. Increases in GDP and OGCN lead to higher CO_2_ emissions, and more stringent environmental policies do not seem to guarantee an immediate decrease in CO_2_. Finally, IMP shows path dependence, with more virtuous countries increasing their energy security.

Table 6 shows the variance decomposition (obtained following the Cholesky decomposition using 1000 Monte Carlo simulations for 10 periods), which evaluates the relative importance of shocks in one variable on variations in other variables over time.

**Table 6:** Variance decomposition analysis

Response variable	Impulse variable						
	dREN (%)	dEPS (%)	dGDP (%)	dCO_2_ (%)	dOGCN (%)	dEDU (%)	dIMP (%)
dREN	68.83	1.54	2.79	3.29	20.48	2.73	0.33
dEPS	8.36	67.37	0.50	17.10	6.27	0.25	0.15
dGDP	4.45	8.49	83.49	1.70	0.69	1.03	0.15
dCO_2_	16.49	11.68	27.34	38.43	2.16	3.76	0.14
dOGCN	68.09	2.00	1.93	3.70	20.78	3.30	0.20
dEDU	0.87	12.04	4.55	3.11	3.52	73.73	2.18
dIMP	12.34	11.40	0.87	18.87	7.83	0.65	48.04

Variation in response variable explained by the impulse variables in the columns (10 periods ahead)

The table highlights that usually each variable is mainly influenced by its lag. Specifically, REN is mainly determined by OGCN (20.48%), CO_2_ (3.29%), and GDP (2.79%) on average during a 10-year period, while OGCN is mainly influenced by shocks in REN (68.09%). An increase in REN could impact on the production of energy from traditional sources at an early stage, and this can negatively affect REN in the future, although this effect is gradual and indirect.

**Fig. 1:**
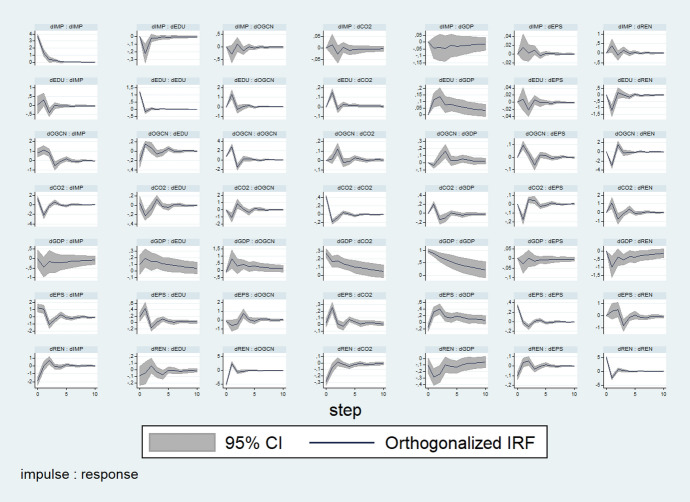
Impulse response analysis

**Fig. 2:**
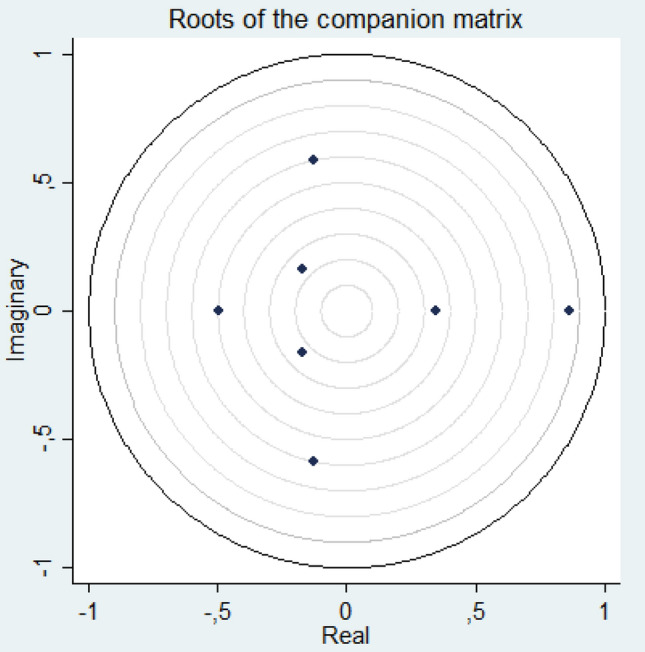
Roots of the companion matrix

Figure 1 illustrates the impulse response functions, which describe the evolution of a variable of interest along the time horizon after a shock in another variable.

When one positive unit shock is applied to one variable in the current period, the response variable usually exhibits a remarkable response in the early phases, followed by a slight fluctuation. Interestingly, when a positive shock is exerted on GDP or OGCN in the present period, REN exhibits negative responses in early phases.

### High and low energy importers

The European Green Deal, in setting objectives in terms of renewable sources development (even for those countries that are rich in traditional energy sources), stresses that countries move from different starting points and are likely to need different strategies. Our intention is to consider these diverse initial conditions. In this context, some scholars have advanced the hypothesis that there exists a relationship between energy security and REP (Chien & Hu, 2008; Gan et al., 2007; Marques & Fuinhas, 2011). Others have suggested that in order to appreciate a country’s efforts towards REP, it might be useful to consider the actual level of installed capacity (Bourcet, 2020). We decided to split the sample into two groups, based on recent energy imports; in fact, this reflects the (current) different stages of countries in terms of levels of installed capacity and energy security.

We examine the role of institutional and socio-technical variables in the production of renewable energy for two panels (A and B) of 10 and 8 EU member states that are, respectively, high energy importers with a share from third countries higher than 50% on average over the last five years (Austria, Belgium, Germany, Greece, Hungary, Ireland, Italy, Portugal, Slovak Republic, and Spain) and low energy importers with a share less than 50% on average (Czech Republic, Denmark, Finland, France, Netherlands, Poland, Sweden, and United Kingdom). The choice is rational and does not create any distortion for the analysis: the two groups would have been substantially the same even if we had used the average percentage of energy imports over the entire period under consideration (with the only exception being Finland, which would have switched from one panel to the other). Similarly, if we had used a different threshold such as the median IMP, over the past 5 years (56.77%) or for the entire period under consideration (58.34%), we would have obtained a similar split: only Hungary would have moved from one sub-sample to the other, based on its average IMP (also in this case calculated over the last 5 years or over the entire period). Moreover, based on Table 2, the negative minimum value of IMP means that some countries have been net exporters for some years. Specifically, UK was mostly a net exporter in the 1990s, while Denmark was a net exporter in the 2000s.

We again employed the PVAR approach. To limit the length of the analysis we opted to include in Appendix 2 the results of some preliminary analyses: the descriptive statistics (see Tables 13 and 14), the unit root tests for both variables in level and in first differences (see Tables 15, 16, 17, and 18), the cointegration tests (see Tables 19 and 20), and the lag order selection analyses (see Tables 21 and 22).

All coefficients resulting from the PVAR analyses conducted over both Panels A and B are as shown in Tables 7 and 8.

**Table 7:** PVAR results—panel A

Independent variables	Dependent variables					
	dREN	dEPS	dGDP	dCO_2_	dOGCN	dEDU
dREN	3.759***	− 0.200***	0.131***	− 0.103	− 4.053***	0.213***
dEPS	26.516***	− 0.247***	1.537***	0.154	− 26.414***	2.502***
dGDP	3.287***	− 0.001	0.645***	0.194***	− 3.236***	0.529***
dCO_2_	− 11.504***	0.084	1.008***	0.158	11.430***	− 1.154***
dOGCN	4.423***	− 0.199***	0.095**	− 0.118*	− 4.716***	0.240***
dEDU	0.079	− 0.026*	0.014	0.047***	− 0.089	0.329***

*p < 0.1; **p < 0.05; ***p < 0.01

**Table 8:** PVAR results—panel B

Independent variables	Dependent variables					
	dREN	dEPS	dGDP	dCO_2_	dOGCN	dEDU
dREN	− 0.380**	0.015**	− 0.168***	− 0.189***	0.503***	0.234***
dEPS	− 8.525***	− 0.150***	1.584***	0.940***	7.530***	0.666***
dGDP	− 1.804***	0.018	0.633***	0.147***	1.564***	0.163***
dCO_2_	2.953***	− 0.217***	0.279***	− 0.486***	− 2.771***	− 0.357***
dOGCN	− 0.548***	0.027***	− 0.124***	− 0.120***	0.592***	0.223***
dEDU	− 0.309**	0.010	0.106***	0.118***	0.490***	− 0.300***

*p < 0.1; **p < 0.05; ***p < 0.01

For the first group of member states (panel A, high energy importers), the results show that policy stringency has positive direct effects on REN. Based on the coefficients, an increase in EPS would also generate a decrease in OGCN. The reduction in OGCN, in turn, does not seem to exert a reduction in REN, probably due to its relatively low weight in the case of high energy importers. This finding does not necessarily mean that OGCN is irrelevant for REN, but rather that the effects of traditional energy industries are not large enough to affect REN. As expected, the analysis shows positive bidirectional casual relationships between GDP and REN. A decrease in CO_2_ also seems to generate an increase in REN. Furthermore, a dynamic effect (path dependence) is evident, demonstrating that the input of GDP and REN observed in previous periods affects that in the present period. If policy stringency, together with income, brings an increase in REN, this will mitigate policy stringency in the long run.

For the second group of countries (panel B, low energy importers), the results show that policy stringency has a negative effect on REN, at least immediately. OGCN negatively affects the transition to renewable energy. As expected, this implies that existing energy production systems, which for electricity are mainly based on oil, natural gas, coal, and nuclear sources, could place a substantial burden on the deployment of renewable energy. Therefore, in places where core energy infrastructures still depend heavily on non-renewable sources, the traditional sector represents the reference point governments should focus upon in their efforts to promote REN. Fostering a renewable-energy economy will probably require a longer period. As expected, an increase in CO_2_ and public awareness will promote an increase in REN. Furthermore, an increase in REN could generate a positive reaction in terms of OGCN, and lobbying could once again slow the transition to a renewable-based economy.

Furthermore, the results show the existence of dynamic effects in terms of GDP and OGCN. As expected, there was a negative causal relationship between REN and CO_2_ for both groups. This implies that the transition to renewable energy should mitigate carbon dioxide emissions and their negative consequences in terms of both climate change and global warming. Finally, our findings show that EDU does not significantly influence REN in the case of panel A, but influences it negatively in the case of panel B.

Similar results have been obtained both by splitting the sample on the median IMP, and by excluding from the analysis those countries that in the past were net exporters.

**Fig. 3:**
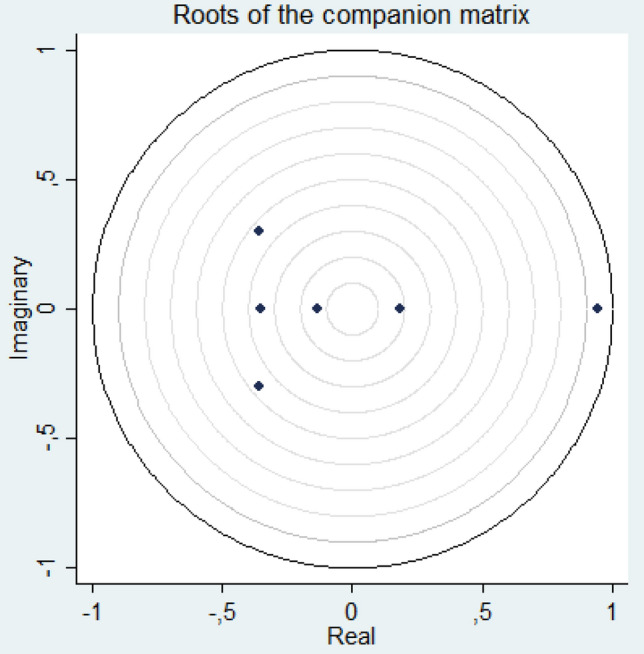
Roots of the companion matrix—panel A

**Fig. 4:**
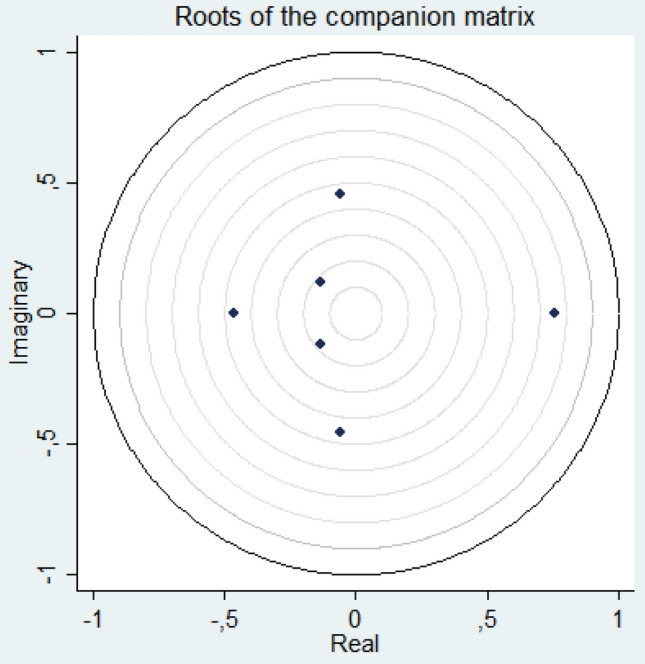
Roots of the companion matrix—panel B

**Fig. 5:**
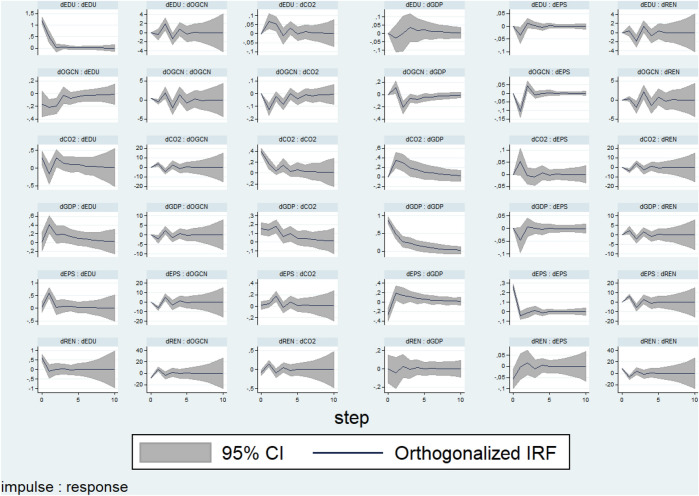
Impulse response analysis—panel A

**Fig. 6:**
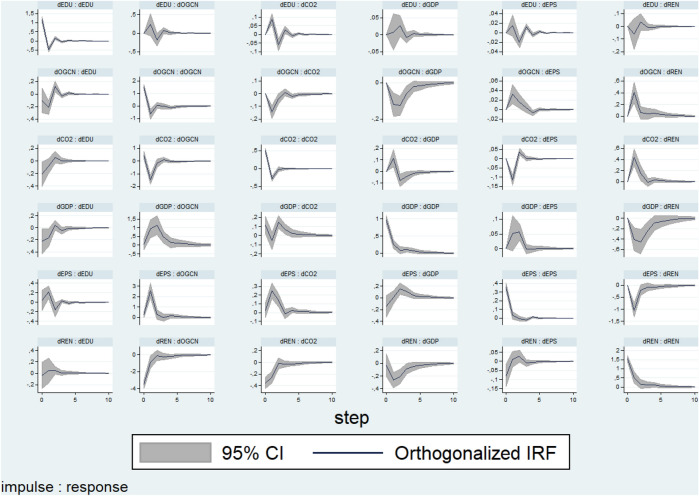
Impulse response analysis—panel B

Appendix 2 also contains the results of the stability analyses (see Tables 23 and 24, and Figs. 3 and 4), the Granger causality tests (see Tables 25 and 26), the variance decomposition analysis (see Tables 27 and 28), and the impulse response analyses (see Figs. 5 and 6).

## Conclusions and policy implications

This article contributes to the debate on the determinants of REP, focusing on the role that institutional and socio-technical aspects play, an aspect which has been considered only partially in the literature (Bourcet, 2020). We employed a PVAR model in first differences to test the complex dynamic relationships among REP, policy stringency, public awareness, lobbying, and education, controlling for per capita income and share of energy imports, for 18 European Union countries.

The results show that EPS does not impact significantly on REN, at least in the short run. This is probably due to the strong heterogeneity among countries, which are different in terms of the level of installed capacity and energy security. An increase in GDP has a negative effect on REN: economic growth is related to an increase in energy demand, and the development of renewable energy sources is often not enough to meet this higher demand. This seems to be confirmed by the fact that an increase in GDP is followed by an increase in energy consumption from traditional energy sources (OGCN increases, with further negative repercussions on REN).

An increase in CO_2_ and public awareness would promote an increase in REN. EDU would impact negatively on REN. This does not mean that education may represent a barrier to the transition to REP. Countries with higher levels of education usually adopt models of production and consumption that inevitably need more energy; as already highlighted, a higher need for energy has often been met through a more vigorous use of traditional sources, especially in past decades. Education cannot compensate for such high levels of energy demand. Moreover, despite the importance of public awareness in the conversion process, stakeholders might not exert enough pressure to encourage a move towards renewable sources.

According to the recent European Green Deal which emphasizes that countries starting from different points need different strategies, we split the entire sample into two panels (A and B) of 10 and 8 EU member states that currently are high and low energy importers, respectively. This allowed us to consider the starting points for countries that are different in terms of level of installed capacity and energy security.

With regard to panel A, estimations show that policy stringency has positive direct effects on REP and should be prioritized to promote the transition to a renewable-energy-based economy. This finding is in line with the existing literature concerning the need for public intervention to promote renewable energy use. As a result, the existence of support policies is expected to have a positive influence on REP. Based on the coefficients, an increase in stringency will generate a decrease in lobbying: these results, although predictable, have rarely been highlighted in the literature (Bourcet, 2020). As expected, the analysis shows the existence of positive bidirectional casual relationships between income and REP. Because stringency positively affects income, policymakers should have more chance to promote renewable sources. A decrease in CO_2_ emissions also seems to generate an increase in REP. The results suggest that if policy stringency, together with public awareness and income, leads to an increase in REP, this will mitigate policy stringency in the long run. Therefore, to support REP, policy efforts must be well established and consistently preserved if there are increasing returns to exploit.

For panel B, the results show that lobbying negatively affects the transition to REP. As expected, this implies that existing energy production systems, primarily based on oil, natural gas, coal, and nuclear sources for the generation of electricity, could represent a substantial burden on the deployment of renewable energy. Fostering a renewable-energy economy will probably require a longer period. If governments decide to continue to invest in traditional sources, this might represent a further obstacle to the growth of REP. Furthermore, an increase in REP could generate a positive reaction in terms of lobbying, and lobbying could once again slow the transition to a renewable-based economy. In this case, to promote the transition to REP, priority should be given to social efforts to decrease the share of traditional energy sources. The analysis shows the existence of dynamic effects in terms of income and lobbying. Given the negative effects of income and lobbying on REP, policymakers should increase their efforts to reduce the proportion of electricity generated from non-renewable energy, and initiate an ambitious process that will ensure progress in the transition to REP in the long term. In other words, the efforts should be followed by policies that ensure a constant commitment to renewable sources (inducing eco-friendly activities; developing market conditions that can create a massive demand for renewable energy use and renewable energy technologies and products).

As expected, there was a negative causal relationship between REP and CO_2_ emissions for both the groups. Informal pressures come mainly from the public and relate to social norms, and to values and expectations that must be respected. The public plays a significant role by providing awareness of REP and influencing the policies and procedures of other actors. On the contrary, the relationship could also reflect society’s apathy towards environmental problems.

The analysis shows that countries starting from different initial conditions in terms of installed capacity, need different transition policies. Data show that historically high/low energy importing countries have remained such over the years: the starting point becomes a country-specific condition for the transition process towards a greener economy. The split between the two panels is substantially independent on the type of threshold used (IMP equal to 50% or its median value) and on the period observed—whole or limited to the last 5 years. To sum up, policymakers should direct their efforts in different directions, bearing in mind two important aspects. First, there are strong divergences that derive from the countries’ initial starting points because of differing economic and socio-political contexts. More feasible action is needed, especially for those countries that are low importers (this may represent an obstacle with regard to initiating a more vigorous transition to renewable energy). Secondly, policy efforts toward REP need continuity as REP is a dynamic process with time lags. National governments should increase the level of education and public pressure by working with schools, training institutions, and universities to prepare the stakeholders who will ensure continuity in the transition toward renewable energy. In short, policymakers should have the vision and patience to understand how yesterday’s choices affect today’s results.

Although our focus has been on the EU, it might be interesting to replicate the exercise with regard to US federal states to appreciate the impact that socio-technical drivers produce on energy transition, net of the policies adopted over time. As seen above, some analyses have been proposed for the US, all emphasizing the positive effects that policies and regulation have on renewable energy, but our model would allow us to appreciate the extent to which socio-technical aspects prop up the policies adopted by state governments.

## Appendix 1

See Tables 9, 10, 11, 12 and Fig. 2.

**Table 9:** Cointegration tests

Statistic	Value	p-value
G_τ_	− 0.652	1.000
G_α_	− 0.819	1.000
P_τ_	− 2.823	0.920
P_α_	− 0.963	0.940

p-value are robust critical values obtained through bootstrapping with 100 replications

**Table 10:** Lag order selection criteria

Lag	MBIC	MAIC	MQIC
1	− 401.261	− 61.391	− 198.380
2	− 214.099	− 44.164	− 112.659

**Table 11:** Eigenvalue stability condition

Eigenvalue		
Real	Imaginary	Modulus
0.860	0.000	0.860
− 0.126	0.586	0.600
− 0.126	− 0.586	0.600
− 0.494	0.000	0.494
0.344	0.000	0.344
− 0.174	− 0.161	0.237

**Table 12:** Granger causality test

Equation Variable	Excluded Variables	Chi2	p value
dREN	dEPS	0.773	0.379
	dGDP	46.827	0.000
	dCO_2_	4.286	0.038
	dOGCN	123.386	0.000
	dEDU	24.778	0.000
	dIMP	4.371	0.037
	ALL	160.018	0.000
dEPS	dREN	37.300	0.000
	dGDP	18.627	0.000
	dCO_2_	55.335	0.000
	dOGCN	43.451	0.000
	dEDU	0.308	0.579
	dIMP	1.105	0.293
	ALL	80.288	0.000
dGDP	dREN	0.064	0.801
	dEPS	79.738	0.000
	dCO_2_	45.498	0.000
	dOGCN	0.042	0.837
	dEDU	18.392	0.000
	dIMP	1.238	0.266
	ALL	224.276	0.000
dCO_2_	dREN	2.018	0.155
	dEPS	103.237	0.000
	dGDP	64.674	0.000
	dOGCN	3.525	0.060
	dEDU	73.701	0.000
	dIMP	0.361	0.548
	ALL	203.351	0.000
dOGCN	dREN	148.024	0.000
	dEPS	0.431	0.511
	dGDP	35.738	0.000
	dCO_2_	5.868	0.015
	dEDU	28.630	0.000
	dIMP	2.469	0.116
	ALL	180.992	0.000
dEDU	dREN	35.446	0.000
	dEPS	55.309	0.000
	dGDP	20.679	0.000
	dCO_2_	6.411	0.011
	dOGCN	50.939	0.000
	dIMP	13.596	0.000
	ALL	117.404	0.000
dIMP	dREN	20.346	0.000
	dEPS	26.963	0.000
	dGDP	34.910	0.000
	dCO_2_	87.331	0.000
	dOGCN	28.371	0.000
	dEDU	5.025	0.025
	ALL	164.845	0.000

## Appendix 2

See Tables 13, 14, 15, 16, 17, 18, 19, 20, 21, 22, 23, 24, 25, 26, 27, 28 and Figs. 3, 4, 5, 6.

**Table 13:** Descriptive statistics—panel A

Variable	Mean	Std. Dev	Min	Max
REN	20.63	19.98	0.58	81.06
EPS	1.76	0.81	0.35	3.33
GDP	29.71	12.20	7.71	65.43
CO_2_	7.74	1.87	4.24	11.62
OGCN	77.92	19.66	17.72	99.28
EDU	58.74	17.56	19.30	85.40

**Table 14:** Descriptive statistics—panel B

Variable	Mean	Std. Dev	Min	Max
REN	17.28	17.42	1.10	65.51
EPS	1.97	1.00	0.54	4.13
GDP	35.92	15.23	5.51	61.18
CO_2_	8.91	2.31	4.48	13.72
OGCN	80.91	18.39	31.90	98.75
EDU	71.82	8.26	47.50	87.60

**Table 15:** Unit root tests: variables in level—panel A

Variable	IPS W-t-bar	MW	Pesaran Z-t-bar
REN	9.660	10.449	1.430
EPS	2.436	8.536	1.121
GDP	1.859	8.815	− 0.186
CO_2_	3.843	3.406	− 1.255
OGCN	8.552	9.471	0.495
EDU	0.993	10.261	− 0.915

*p < 0.1; **p < 0.05; ***p < 0.01

**Table 16:** Unit root tests: variables in level—panel B

Variable	IPS W-t-bar	MW	Pesaran Z-t-bar
REN	8.155	3.030	2.757
EPS	2.892	2.360	− 1.519*
GDP	1.733	7.487	0.013
CO_2_	2.771	4.642	− 1.661**
OGCN	7.277	3.228	2.215
EDU	1.037	9.595	1.163

*p < 0.1; **p < 0.05; ***p < 0.01

**Table 17:** Unit root tests: variables in first differences—panel A

Variable	IPS W-t-bar	MW	Pesaran Z-t-bar
dREN	− 8.489***	133.825***	− 3.708***
dEPS	− 10.938***	105.490***	− 3.254***
dGDP	− 6.0277***	82.309***	− 0.359
dCO_2_	− 10.861***	75.727***	− 6.921***
dOGCN	− 8.776***	135.399***	− 3.874***
dEDU	− 10.835***	190.356***	− 4.912***

*p < 0.1; **p < 0.05; ***p < 0.01

**Table 18:** Unit root tests: variables in first differences—panel B

Variable	IPS W-t-bar	MW	Pesaran Z-t-bar
dREN	− 7.216***	90.254***	− 3.432***
dEPS	− 12.453***	99.252***	− 5.722***
dGDP	− 7.032***	62.763***	− 1.858**
dCO_2_	− 13.891***	102.513***	− 6.441***
dOGCN	− 8.014***	107.788***	− 4.016***
dEDU	− 11.420***	97.991***	− 6.858***

*p < 0.1; **p < 0.05; ***p < 0.01

**Table 19:** Cointegration tests—panel A

Statistic	Value	p-value
G_τ_	− 0.536	1.000
G_α_	− 0.69	1.000
P_τ_	− 1.06	0.950
P_α_	− 0.151	0.970

p-value are robust critical values obtained through bootstrapping with 100 replications

**Table 20:** Cointegration tests—panel B

Statistic	Value	p-value
G_τ_	− 0.466	0.990
G_α_	− 0.296	1.000
P_τ_	− 1.447	0.940
P_α_	− 0.684	0.930

p-value are robust critical values obtained through bootstrapping with 100 replications

**Table 21:** Lag order selection criteria—panel A

Lag	MBIC	MAIC	MQIC
1	− 266.807	− 59.245	− 143.589
2	− 128.755	− 24.974	− 67.146

**Table 22:** Lag order selection criteria—panel B

Lag	MBIC	MAIC	MQIC
1	− 260.540	− 77.417	− 151.622
2	− 114.614	− 27.033	− 62.523

**Table 23:** Eigenvalue stability condition—panel A

Eigenvalue		
Real	Imaginary	Modulus
0.945	0.000	0.945
− 0.356	0.298	0.464
− 0.356	− 0.298	0.464
− 0.352	0.000	0.352
0.181	0.000	0.181
− 0.136	0.000	0.136

**Table 24:** Eigenvalue stability condition—panel B

Eigenvalue		
Real	Imaginary	Modulus
0.755	0.000	0.755
− 0.461	0.000	0.461
− 0.060	− 0.455	0.459
− 0.060	0.455	0.459
− 0.132	− 0.116	0.176
− 0.132	0.116	0.176

**Table 25:** Granger causality test—panel A

Equation variable	Excluded variables	Chi2	p value
dREN	dEPS	26.340	0.000
	dGDP	8.872	0.003
	dCO_2_	12.058	0.001
	dOGCN	20.169	0.000
	dEDU	0.022	0.883
	ALL	41.662	0.000
dEPS	dREN	31.785	0.000
	dGDP	0.002	0.961
	dCO_2_	2.056	0.152
	dOGCN	30.939	0.000
	dEDU	3.650	0.056
	ALL	45.037	0.000
dGDP	dREN	10.672	0.001
	dEPS	48.417	0.000
	dCO_2_	16.282	0.000
	dOGCN	5.648	0.017
	dEDU	0.185	0.667
	ALL	60.787	0.000
dCO_2_	dREN	2.083	0.149
	dEPS	2.656	0.103
	dGDP	32.588	0.000
	dOGCN	2.710	0.100
	dEDU	7.417	0.006
	ALL	97.506	0.000
dOGCN	dREN	16.641	0.000
	dEPS	26.304	0.000
	dGDP	8.648	0.003
	dCO_2_	11.991	0.001
	dEDU	0.027	0.869
	ALL	40.769	0.000
dEDU	dREN	7.369	0.007
	dEPS	35.266	0.000
	dGDP	21.782	0.000
	dCO_2_	9.286	0.002
	dOGCN	9.482	0.002
	ALL	46.319	0.000

**Table 26:** Granger causality test—panel B

Equation variable	Excluded variables	Chi2	p value
dREN	dEPS	82.194	0.000
	dGDP	77.824	0.000
	dCO_2_	52.848	0.000
	dOGCN	13.614	0.000
	dEDU	5.323	0.021
	ALL	167.613	0.000
dEPS	dREN	3.276	0.070
	dGDP	1.323	0.250
	dCO_2_	102.414	0.000
	dOGCN	10.419	0.001
	dEDU	1.715	0.190
	ALL	152.624	0.000
dGDP	dREN	26.495	0.000
	dEPS	96.287	0.000
	dCO_2_	17.812	0.000
	dOGCN	12.767	0.000
	dEDU	28.473	0.000
	ALL	268.514	0.000
dCO_2_	dREN	46.566	0.000
	dEPS	89.081	0.000
	dGDP	28.202	0.000
	dOGCN	19.274	0.000
	dEDU	76.066	0.000
	ALL	566.768	0.000
dOGCN	dREN	11.613	0.001
	dEPS	82.795	0.000
	dGDP	77.447	0.000
	dCO_2_	65.287	0.000
	dEDU	16.727	0.000
	ALL	196.342	0.000
dEDU	dREN	84.425	0.000
	dEPS	23.436	0.000
	dGDP	13.619	0.000
	dCO_2_	31.347	0.000
	dOGCN	97.284	0.000
	ALL	225.449	0.000

**Table 27:** Variance decomposition analysis—panel A

Response variable	Impulse variable					
	dREN (%)	dEPS (%)	dGDP (%)	dCO_2_ (%)	dOGCN (%)	dEDU (%)
dREN	53.48	25.40	0.39	11.33	8.25	1.14
dEPS	4.96	83.53	0.25	1.50	8.65	1.10
dGDP	0.29	7.17	86.72	5.23	0.55	0.04
dCO_2_	9.26	5.81	53.75	28.34	1.87	0.96
dOGCN	53.37	25.15	0.41	11.26	8.69	1.14
dEDU	11.14	10.38	9.82	4.85	2.09	61.72

Variation in response variable explained by the impulse variables in the columns (10 periods ahead)

**Table 28:** Variance decomposition analysis—panel B

Response variable	Impulse variable					
	dREN (%)	dEPS (%)	dGDP (%)	dCO_2_ (%)	dOGCN (%)	dEDU (%)
dREN	59.70	22.66	9.54	6.01	1.52	0.56
dEPS	5.25	84.69	0.65	7.46	1.21	0.73
dGDP	19.22	12.63	65.14	1.68	0.58	0.75
dCO_2_	33.86	13.64	9.56	35.92	3.48	3.54
dOGCN	57.47	21.80	6.97	6.47	5.78	1.51
dEDU	3.04	4.12	0.44	2.04	3.41	86.96

Variation in response variable explained by the impulse variables in the columns (10 periods ahead)
